# Fast and Selective Aqueous-Phase Oxidation of Styrene to Acetophenone Using a Mesoporous Janus-Type Palladium Catalyst

**DOI:** 10.3390/molecules26216450

**Published:** 2021-10-26

**Authors:** Majid Vafaeezadeh, Ranja Saynisch, Andrea Lösch, Wolfgang Kleist, Werner R. Thiel

**Affiliations:** Fachbereich Chemie, Technische Universität Kaiserslautern, Erwin-Schrödinger-Str. 54, D-67663 Kaiserslautern, Germany; saynisch@rhrk.uni-kl.de (R.S.); loesch@chemie.uni-kl.de (A.L.); kleist@chemie.uni-kl.de (W.K.)

**Keywords:** mesoporous Janus-type catalyst, modified mesoporous silica, aqueous-phase oxidation, palladium catalyst, hydrogen peroxide

## Abstract

A heterogeneous Janus-type palladium interphase catalyst was obtained by selective surface modification of a hollow mesoporous silica material. The catalyst comprises hydrophobic octyl groups on one side of the silica nanosheets and single-site bis-imidazoline dichlorido palladium(II) complexes on the other. The structure of this composite material has been analyzed by means of elemental analysis, atomic absorption spectroscopy, BET surface analysis, TGA, SEM and solid-state CP-MAS ^13^C and ^29^Si NMR spectroscopy. The catalyst showed extraordinary activity for the aqueous-phase oxidation of styrene to acetophenone using 30% hydrogen peroxide as the oxidant. An 88% yield of acetophenone could be achieved after 60 min.

## 1. Introduction

The Wacker oxidation is an industrial method for the conversion of ethylene to acetaldehyde. It has been performed by employing catalytic amounts of palladium together with stoichiometric amounts of copper salts as the co-catalyst [1]. The procedure is also well known for the oxidation of other terminal olefins [2], allowing for the preparation of valuable chemicals such as ketones, epoxides or benzaldehyde derivatives using either O_2_ [3,4,5,6], hydrogen peroxide (H_2_O_2_) [7,8,9,10] or *tert*-butyl hydroperoxide (TBHP) [11,12] as the most common oxidants. Among the employed oxidants, H_2_O_2_ can be considered as a less hazardous oxidant since water is the sole by-product of the reaction and the use of copper as co-catalyst is not necessary. Moreover, due to the development of novel technologies during the last decades, the production of H_2_O_2_ is a cheap and safe process [13].

Several homogeneous and heterogeneous palladium catalysts were employed for this reaction in the past [3,4,5,6,7,8,9,10,11,12,14,15,16,17]. While homogeneous palladium catalysts showed rather high efficiencies, they suffer from the poor recovery of the expensive noble metal. Moreover, in some case, the reaction proceeded well only in the presence of expensive organic ligands or additives [18,19,20,21]. In contrast, heterogeneous catalysts allow a simple recovery and separation of the palladium source. However, they encounter a number of limitations for the Wacker oxidation: the reaction rate of the heterogeneous catalyst is lower, and hence, longer reaction times are required to achieve desirable conversions. Increasing the reaction time may deactivate the palladium centers on the surface of the applied support and in consequence, request more energy to complete the reaction. Another challenge of a long-time oxidation reaction is related to the gradual decomposition of H_2_O_2_. Therefore, in some cases, up to 10 equiv. of the oxidant had to be added [9], which is not a cost-effective and sustainable protocol for an oxidation reaction.

A routine method for the synthesis of heterogeneous metal catalysts is the deposition of the metal nanoparticles on the surface of the support. Although this seems to be a fairly simple procedure, it suffers from noticeable leaching of the metal from the surface and undesirable steric effect between the surface and the reactants. In the case of palladium catalysts, this often leads to a rapid termination of the catalyst’s activity via the formation of inactive palladium black species.

By employing heterogeneous interphase catalysts, it was possible to improve the catalytic activity to some extent [22]. In interphase catalysts, the active centers are connected with covalent bonds to the surface via an organic spacer and hence, are in principle far away from the surface’s steric effects. This method also allows the immobilization of site-isolated metal complexes. However, compared to homogeneous catalysts, the efficiency of such catalysts is still far from a desirable threshold.

Janus-type materials are anisotropic double-face architectures, which have successfully been introduced as a new material generation for heterogeneous catalysis [23]. Due to their unique properties, Janus-type catalysts can bridge the gap between homo- and heterogeneous catalysts by combination of the activity of homogeneous systems and the simple separation and storage of the heterogeneous ones.

Recently, we established the concept of “Janus interphase catalysts” [24]. In this type of solid catalyst, the surface polarity can be manipulated by incorporation of in principle any type of organic group having a specific polarity with the aim of improving the mass transfer of the substrates to the active centers. Consequently, heterogeneous Janus-type interphase catalysts with overall higher activities compared to the classical heterogeneous catalysts have been generated [25,26,27,28].

To this aim, we introduced simple and practical techniques for the synthesis of heterogeneous Janus-type interphase catalysts by a modification of the Stöber process [29,30,31]. The catalysts showed high activity for the synthesis of a series of fine chemicals. The presence of water either as the solvent or as a by-product has no negative effects on the reaction efficiency. In order to extend this concept, we wish to disclose the first Janus-type interphase palladium catalyst and its applications for the Wacker-type oxidation of styrene to acetophenone employing H_2_O_2_ as the oxidant. Thanks to the unique structure of the material, it shows higher activity even in comparison to a homogeneous PdCl_2_–based system.

## 2. Results and Discussion

**Scheme 1:**
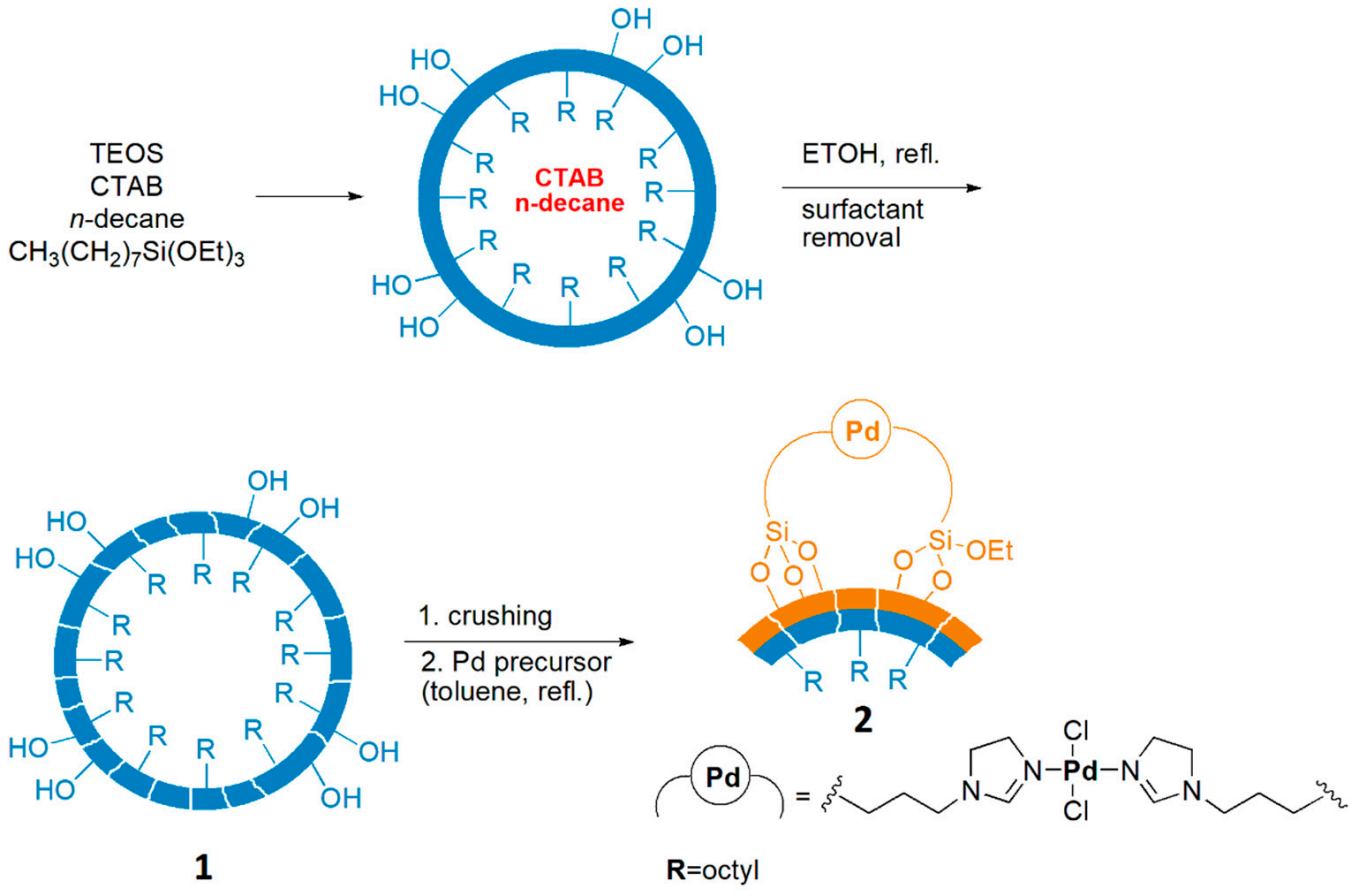
Schematic synthesis of the heterogeneous Janus-type Pd interphase catalyst **2**.

The material was prepared from commercially available starting materials (Scheme 1). In the first step, hollow spheres with octyl groups (R) that are covalently immobilized on the inner surface were prepared by using a modified Stöber method for the preparation of monodisperse silica particles followed by removing of the surfactant to yield the mesoporous hollow structure **1**. After crushing the hollow particles, the palladium precursor was grafted to the surface to form the final catalyst **2**.

The loading of palladium in the catalyst was determined based on an atomic absorption spectroscopy (AAS) to be 0.22 mmol∙g^–1^.

**Figure 1:**
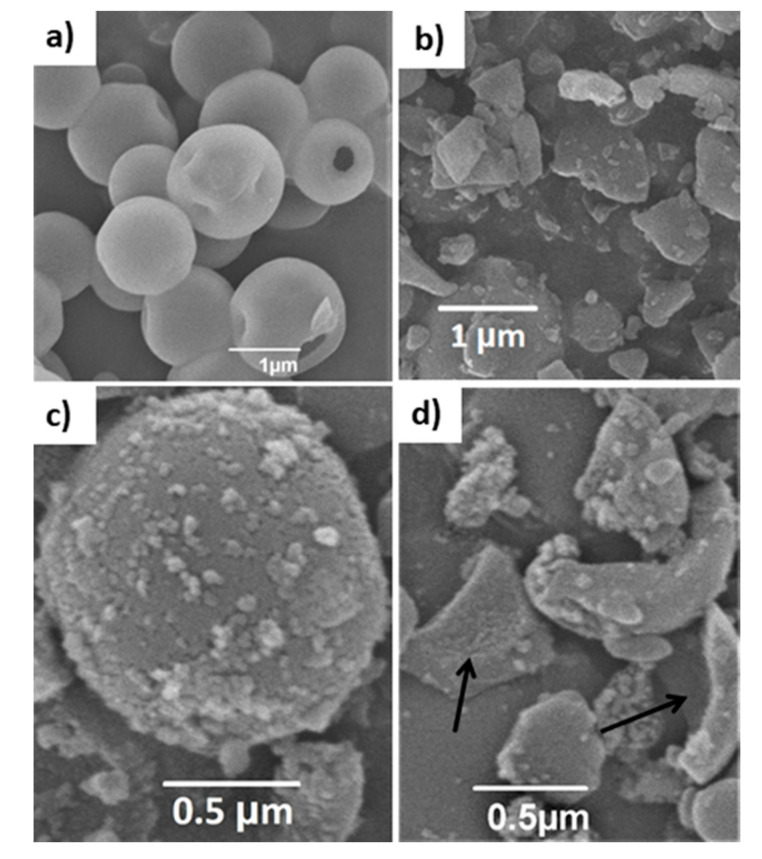
(**a**) SEM images of the hollow spheric material **1**, (**b**) nanosheets of the final catalyst **2**, (**c**) outer surface of catalyst **2** after the labelling experiment and (**d**) the inner surface of catalyst **2** after the labelling experiment.

Scanning electron microscope (SEM) images of material **1** and **2** are presented in Figure 1. Material **1** shows the expected geometry with rather monodisperse spheres of approximately 1 μm in diameter (Figure 1a). After crushing, nanosheets of catalyst **2** were formed (Figure 1b). To find evidence for a selective surface functionalization of catalyst **2**, the material in the middle of crushing process was labelled with negatively charged citrate-capped Fe_3_O_4_ nanoparticles (Fe_3_O_4_@citrate) according to our previously described procedure [30].

After labelling the catalyst by mixing it with citrate@Fe_3_O_4_, the outer surface of the material that contains the nitrogen ligand can interact with the labelling agent (Figure 1c), while due to lack of noticeable functionality of the inner surface (i.e., relatively inert octyl groups), it remained almost remains untouched (Figure 1d).

**Figure 2:**
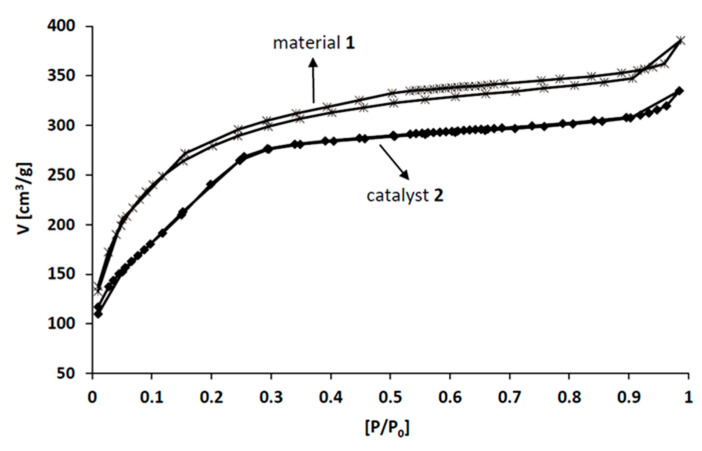
N_2_ adsorption/desorption isotherms of material **1** and catalyst **2**.

The N_2_ adsorption/desorption isotherms of materials **1** and catalyst **2** (Figure 2,) show a type IV profile, which is typical for mesoporous materials. The calculated BET surface areas, the average pore diameters (BJH method) and the pore volumes for the hollow material **1** are 1045 m^2^∙g^–1^, 2.29 nm and 0.60 mL∙g^–1^, while the corresponding values for the final catalyst **2** are 848 m^2^∙g^–1^, 2.45 nm and 0.52 mL∙g^–1^, respectively. This observation suggests that after the functionalization of material **1**, the specific surface area as well as pore volume in the final crushed catalyst **2** are reduced, which is reasonable evidence for the successful functionalization.

**Figure 3:**
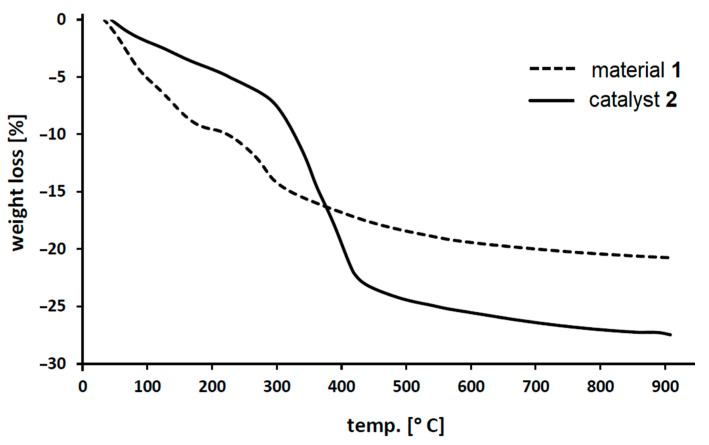
TGA of material **1** and catalyst **2**.

Comparative thermogravimetric analysis (TGA) results for materials **1** and **2** are presented in Figure 3. It can be deduced from this thermogram that catalyst **2** is stable up to 300 °C. A small weight loss (~5%) was found by heating the sample up to this temperature, which is attributed to the removal of physisorbed water. However, the presence of water in material **1** is approximately 15%, which indicates the higher affinity of material **1** to water due to the presence of -OH groups on the outer surface. Consequently, catalyst **2** has a balanced hydrophobicity/hydrophilicity and is suitable for aqueous phase oxidation reactions of organic compounds [32].

**Figure 4:**
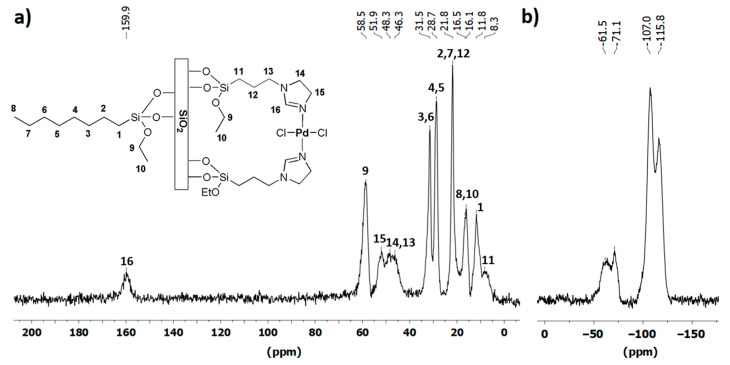
(**a**) Solid-state ^13^C and (**b**) ^29^Si CP-MAS NMR spectra of catalyst **2**.

The nature of the surface functionalities of catalyst **2** was investigated in more detail by means of solid-state CP-MAS ^13^C NMR spectroscopy (Figure 4a). Directly bound to the silicon atoms, the carbon atoms C1 and C11 are highly shielded and are assigned to the resonances at 8.3 and 11.8 ppm, respectively. The methyl groups (carbon atoms C8 and C10) provide resonances at 16.1 and 16.5 ppm. Due to their similar chemical shifts, carbon atoms C2, C7 and C12 give rise to an intense resonance at 21.8 ppm. Two sharp and intense resonances at 28.7 and 31.5 ppm are assigned to carbon atoms C4 and C5 as well as C3 and C6, respectively. Although carbon atoms C13–15 have similar chemical shifts, they can fairly be distinguished in the region from 46.3 to 51.9 ppm. The resonance of some residual ethoxy groups coming from the grafted octyl groups as well as from the palladium complex precursors is observed at 58.5 ppm and the resonance of the unique sp^2^-hybridized carbon atom C16 is observed at 159.9 ppm.

The ^29^Si CP-MAS NMR spectrum of catalyst **2** (Figure 4b, right side) exhibits two dominant resonances at –107 and –116 ppm, being attributed to Q^3^ [Si(OSi)_3_OH] resp. Q^4^ [Si(OSi)_4_] species in the framework of the materials. The presence of Q^3^ signal in the solid-state ^29^Si NMR is evidence for the presence of free hydroxyl (-OH) groups, and hence, a partial hydrophilicity of catalyst **2** even after the second functionalization. Two further broad resonances in this spectrum at around −62 and −71 ppm can be assigned to T^2^ [RSi(OSi)_2_(OH)] and T^3^ [RSi(OSi)_3_] units and thus provide further evidence for the successful covalent immobilization of the palladium precursors on the surface of the materials.

The activity of catalyst **2** was tested for the oxidation of styrene leading to acetophenone by employing 30% aq. H_2_O_2_. Unlike for other alkenes, controlling the selectivity for the oxidation of styrene is more difficult since beside the formation of acetophenone, benzaldehyde and styrene oxide are frequently found. By screening the effect of solvents, the best activity was observed in a mixture of acetic acid and water (1.4:0.6 mL). The results of the styrene oxidation using Janus-type catalyst **2** as well as a comparison with other catalytic systems are summarized in Table 1.

To clarify a possible formation of peroxyacetic acid from the reaction of AcOH and H_2_O_2_ and its effect on the reaction, first, we studied a blank experiment in the absence of any palladium catalyst. No yield of acetophenone was observed after 1 h at 80 °C by employing 3 equiv. of 30% aq. H_2_O_2_ (Table 1, entry 1). These conditions led to the formation of a small amount of benzaldehyde. By addition of 1 mol% of palladium dichloride as the simplest example of a Pd–based catalyst, the yield of the acetophenone reached 67% accompanied by 8% of benzaldehyde as the major by-product (Table 1, entry 2). Surprisingly, 76% of acetophenone was found after 30 min when 45 mg of **2** (1 mol% equiv. of Pd) was used as the catalyst (Table 1, entry 3). This indicates that the activity of catalyst **2** is even higher than the activity of the PdCl_2_–based system providing rather identical selectivity. When the reaction time was increased to 1 h, 88% of acetophenone were isolated (Table 1, entry 4). The reaction in pure acetic acid (2 mL) also afforded good yields of acetophenone. However, it was lower than in the acetic acid/H_2_O system and worse in selectivity (Table 1, entry 5). The possibility of a Wacker oxidation using O_2_ as oxidant was also investigated (Table 1, entry 6). It was found that the reaction can proceed well in the presence of CuCl as a co-catalyst but without a desirable selectivity for acetophenone.

*tert*-Butyl hydroperoxide (TBHP) is also known as an oxidant being suitable for the oxidation of styrene to styrene oxide [11,12]. However, no acetophenone at all was found when TBHP (70% aq.) was used as the oxidant in the presence of catalyst **2**. Instead, 47% of styrene oxide accompanied by 37% of benzaldehyde were formed (Table 1, entry 7). Poor yield and selectivity were observed in the case when the urea adduct of hydrogen peroxide (35% UHP) was applied as the oxidant (Table 1, entry 8).

The activity and selectivity of the heterogeneous Janus-type palladium catalyst for the oxidation of styrene was also compared with some reports of homogeneous and heterogeneous systems (Table 1, entries 9–13). As shown in Table 1, some of the reported protocols suffer from high amounts of palladium catalyst with the need for up to 10 mol% of Na_2_PdCl_4_ or 5 mol% of Pd^0^/C (Table 1, entries 9–10). Another drawback is employing a harsh reaction condition such as scCO_2_ at 120 °C, which resulted a poor conversion (Table 1, entry 11).The use of expensive ligands in concentrated H_2_O_2_ as the oxidant (Table 1, entry 12), or the need for applying up to 10 equiv. of the oxidant (Table 1, entry 13), are among the negative issues of the reported protocols. Consequently, the efficiency of the Janus-type catalyst **2** stands among the top of the reported homogeneous and heterogeneous systems in an economic and environmental point of view.

To study the reusability of catalyst **2**, an experiment was set up with 135 mg of the catalyst and 3.0 mmol of styrene. At the end of the first run, it was cooled down to r.t. and the catalyst was separated by centrifugation and decanting of the reaction mixture. It was dried under vacuum and then reused for two subsequent reaction runs. The yields of the acetophenone for the 2nd and the 3rd reaction run were found to be 81% and 78%, respectively, while 122 mg of the catalyst were isolated at the end of the 3rd reaction run. The slight decrease of the yield can be explained by the small loss of catalyst during the recovery process, which means that there is no principal degradation of the catalyst. The elemental analysis of the recovered catalyst revealed the presence of 1.40% of nitrogen (from the ligand) in the catalyst. This value for the fresh catalyst was 1.42%, which indicates very good stability of the Pd complex on the surface.

**Figure 5:**
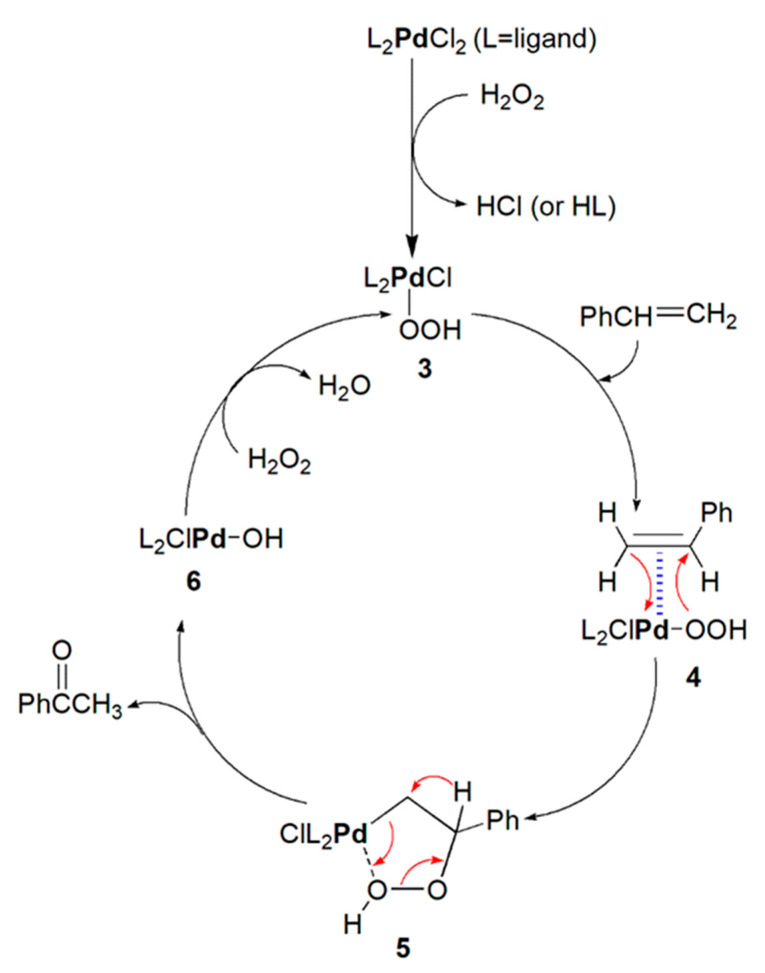
Postulated mechanism for the oxidation styrene to acetophenone.

A proposed reaction mechanism is shown in Figure 5. Our attempt to convert styrene oxide to acetophenone in the presence of an identical amount of catalyst and without employment of any oxidant was not successful. This finding indicates that the reaction cannot proceed via the rearrangement of styrene oxide that is intermediately formed by epoxidation of styrene. In the first step, the reaction of the catalyst and hydrogen peroxide could form the hydroperoxidopalladium intermediate **3** on the surface of the material. The hydrophobic face (decorated with octyl groups) will efficiently accelerate the diffusion of the starting material to the active site. Consequently, styrene and the catalytically active species will meet each other on a nanometric temporary interface and react to produce the Pd π-complex **4** that subsequently converts into the palladadioxa cyclopentane intermediate **5**. This undergoes a 1,2-H shift in combination with a cleavage of the Pd-C and the O-O bond to finally release acetophenone and the hydroxidopalladium species **6**. The reaction cycle then can be continued by the exchange of HO^–^ against HOO^–^.

## 3. Conclusions

In summary, a novel mesoporous Janus-type palladium interphase catalyst was prepared and employed for the Wacker-type oxidation of styrene to acetophenone using 30% H_2_O_2_ as the oxidizing agent. The material was prepared in a simple procedure using cheap starting materials. The catalyst shows excellent activity and selectivity for the synthesis of acetophenone by the oxidation of styrene. The activity and selectivity of the heterogeneous catalyst was found to be superior to a homogeneous PdCl_2_–based system. We assign the main reason for the high activity of this catalyst system to the balanced hydrophobicity/hydrophilicity because of the presence of surface silanol as well as octyl groups on its surface. This combination of functional groups can facilitate the oxidation in a narrow interface where aqueous oxidant and organic substrate meet each other.

## 4. Materials and Methods

### 4.1. General Methods

All chemicals and solvents were purchased from the suppliers and used without further purifications except pentane and toluene, which were dried before use. Solid state ^13^C and ^29^Si CP-MAS NMR spectra were measured using a 500 MHz BRUKER Avance III spectrometer under cross polarization conditions with a spinning frequency of 11,000 Hz for both ^13^C and ^29^Si and with scan numbers of 15,000 and 2500, respectively. TGA measurements were performed in synthetic air (20.5% O_2_ in N_2_) on a SETARAM-Setsys-16 from 30 °C to 900 °C with a heating ramp of 10 K min^−1^. For N_2_ adsorption/desorption, the samples were activated for 24 h at 150 °C prior to the measurements. Adsorption and desorption isotherms were recorded using an Autosorb-1 setup from Quantachrome. The specific surface areas were determined using the BET method. Electron microscopy measurements were conducted on a Jeol JSM-6490LA operated at 15 kV. Prior to the measurements, the samples were sputter-coated with gold (JFC-1200 Fine Coater, Jeol, Tokyo, Japan) for 30 s. High resolution ^1^H and ^13^C NMR analyses were performed with 400 and 600 MHz BRUKER DPX spectrometers. An air-cooled condenser (Findenser^TM^, *SUPER* air condenser, Radleys, Saffron Walden, Essex, UK) was used in the reactions instead of a water-cooled condenser.

### 4.2. Synthesis of Material 1

Material **1** was prepared according to our previously described procedure [29] followed by the crushing of the resulted hollow particles using mortar and pestle for 30 min. Yield: 1.15 g. Elemental analysis: C: 6.72% and H: 2.15%.

### 4.3. Synthesis of the Palladium Precursor


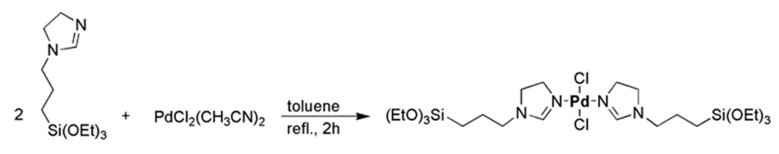


A solution of 0.28 mL (0.28 g, 1.02 mmol) of triethoxy-3-(2-imidazolin-1-yl)propylsilane and 0.130 mg (0.5 mmol) of PdCl_2_(CH_3_CN)_2_ in 20 mL of toluene was heated for 2 h to reflux. At the end of the reaction, the flask was cooled to room temperature and the toluene was evaporated under vacuum. Residual amounts of unreacted silane precursor was washed out with dry pentane (2 × 15 mL). The obtained material was dried under vacuum to afford 327 mg (90%) of the product. ^1^H NMR (600 MHz, CDCl_3_): 7.18 (s, 2H), 3.72–3.82 (m, 16H), 3.25 (t, 4H), 3.08 (t, 4H), 1.54 (quint. 4H), 1.16 (t, 18 H) and 0.47 ppm (t, 4H). ^13^C NMR in CDCl_3_ (151 MHz, CDCl_3_) in ppm: 159.9, 58.5, 53.0, 50.0, 46.9, 21.6, 18.3 and 7.3 ppm. Elemental analysis found: C 40.13, H 6.92, N 7.98, calcd. for C_24_H_52_Cl_2_N_4_O_6_PdSi_2_: C 39.69, H 7.22, N 7.72.

### 4.4. Synthesis of Catalyst 2

In a 50 mL flask equipped with an air condenser, 254 mg (0.35 mmol) of the palladium precursor were dissolved in 20 mL of dry toluene under an atmosphere of N_2_. Then, 1.00 g of the crushed material **1** was added to the flask and the mixture was heated to reflux for 24 h. After cooling to room temperature, the solid was separated by centrifugation, washed several times with acetone and ethanol to remove traces of unreacted palladium precursor, and subsequently dried under vacuum. Yield: 1.18 g. Elemental analysis: C 13.36%, H 2.55%, N 1.42%.

### 4.5. Typical Procedure for Palladium-Catalyzed Oxidation of Styrene Using 30% H_2_O_2_

Measures of 0.11 mL (104 mg, 1 mmol) of styrene, 1.4 mL of acetic acid, 0.6 mL of H_2_O and 0.31 mL (3.0 mmol) of 30% H_2_O_2_ and 45 mg (1.0 mol%) of catalyst **2** were added to a round bottom flask. The mixture was heated to 80 °C for 60 min. At the end, the flask was cooled to room temperature and the catalyst was removed by filtration or centrifuging. The products were purified employing flash a chromatography apparatus with *n*–hexane/ethyl acetate as the solvents. The ratio of ethyl acetate was increased gradually from 0 to 10%. Acetophenone: ^1^H NMR (400 MHz, CDCl_3_): 7.89 (d, 2H), 7.49 (t, 1H), 7.39 (t, 2H) and 2.54 ppm (s, 3H). ^13^C NMR of (101 MHz, CDCl_3_): 198.2, 137.1, 133.1, 128.6, 128.3 and 26.6 ppm.

## Figures and Tables

**Table 1:** A brief comparison of the activities of catalyst **2** in the oxidation of styrene to acetophenone and a comparison with some homogeneous and heterogeneous Pd catalysts used for this transformation (Ap: acetophenone; Ba: benzaldehyde).

Entry	Catalyst (amount)	Oxidant (equiv.)	Time (h)	Solvent	Temp. (°C)	Ap (%)	Ba (%)	Ref.
1	–	30% H_2_O_2_ (3)	1	AcOH/H_2_O ^a^	80	–	12 ^b^	this work
2	PdCl_2_ (1 mol%)	30% H_2_O_2_ (3)	1	AcOH/H_2_O	80	67 ^b^	8 ^b^	this work
3	**2** (1 mol%)	30% H_2_O_2_ (3)	0.5	AcOH/H_2_O	80	76 ^b^	9 ^b^	this work
4	**2** (1 mol%)	30% H_2_O_2_ (3)	1	AcOH/H_2_O	80	88 ^b^	9 ^b^	this work
5	**2** (1 mol%)	30% H_2_O_2_ (3)	1	AcOH	80	82 ^b^	15 ^b^	this work
6	**2** (1 mol%) + CuCl (10 mol%)	O_2_ (balloon)	16	AcOH/H_2_O	80	63 ^b^	30 ^b^	this work
7	**2** (1 mol%)	TBHP ^c^ (2)	16	CH_3_CN	80	–	37 ^b,d^	this work
8	**2** (1 mol%)	35% UHP (3)	16	AcOH/H_2_O	80	47 ^b^	19 ^b^	this work
9	Na_2_PdCl_4_ (10 mol%)	30% H_2_O_2_ (n.d.)	2.5	aq. NMP	r.t.	55	n.d.	[33]
10	Pd^0^/C (5 mol%)	30% H_2_O_2_ (6)	8	CH_3_CN	65	90 ^e^	n.d.	[16]
11	Pd/Al_2_O_3_ (2.5 wt%)	30% H_2_O_2_ (4)	3	scCO_2_	120	83 ^f^	6	[15]
12	(PBO)Pd(CH_3_CN)_2_(OTf)_2_ (1 mol%)	50% H_2_O_2_ (5)	24	CH_3_CN	r.t.	80 ^g^	n.d.	[18]
13	Pd^0^/RGO(0.01 g), GO (0.01 g)	30% H_2_O_2_ (10)	12	CH_3_CN/H_2_O	55	93	n.d.	[9]

^a^ The amounts of AcOH:H_2_O in all reactions were 1.4:0.6 mL. ^b^ Isolated yield. ^c^ 70% solution of TBHP in H_2_O was used. ^d^ 47% of styrene oxide was found. ^e^ 0.2 eq. H_2_SO_4_ was used as an additive. ^f^ 53% conversion of styrene. ^g^ 92% conversion of styrene. Abbreviations: n.d.: not determined; AcOH: acetic acid; UHP: urea hydrogen peroxide; NMP: N-methyl-2-pyrrolidone; scCO_2_: supercritical CO_2_; PBO: 2-(2-pyridyl)benzoxazole; OTf: triflate; RGO: reduced graphene oxide.

## Data Availability

Data is contained within the article.

## Sample Availability

Samples of the compounds are available from the authors.
